# Predictors of Use and Drop Out From a Web-Based Cognitive Behavioral Therapy Program and Health Community for Depression and Anxiety in Primary Care Patients: Secondary Analysis of a Randomized Controlled Trial

**DOI:** 10.2196/52197

**Published:** 2024-01-17

**Authors:** Armando J Rotondi, Bea Herbeck Belnap, Scott Rothenberger, Robert Feldman, Barbara Hanusa, Bruce L Rollman

**Affiliations:** 1 Mental Illness Research Education and Clinical Center VA Pittsburgh Healthcare System Veterans Administration Pittsburgh, PA United States; 2 Center for Behavioral Health, Media and Technology University of Pittsburgh Pittsburgh, PA United States; 3 Center for Health Equity Research and Promotion VA Pittsburgh Healthcare System Veterans Administration Pittsburgh, PA United States; 4 Center for Research on Health Care University of Pittsburgh School of Medicine Pittsburgh, PA United States

**Keywords:** e-mental health, user engagement, initiation, discontinue, depression, anxiety, cognitive behavioral therapy, computerized CBT, online health community, collaborative care, internet support group

## Abstract

**Background:**

A previously reported study examined the treatment of primary care patients with at least moderate severity depressive or anxiety symptoms via an evidence-based computerized cognitive behavioral therapy (CCBT) program (Beating the Blues) and an online health community (OHC) that included a moderated internet support group. The 2 treatment arms proved to be equally successful at 6-month follow-up.

**Objective:**

Although highly promising, e-mental health treatment programs have encountered high rates of noninitiation, poor adherence, and discontinuation. Identifying ways to counter these tendencies is critical for their success. To further explore these issues, this study identified the primary care patient characteristics that increased the chances patients would not initiate the use of an intervention, (ie, not try it even once), initiate use, and go on to discontinue or continue to use an intervention.

**Methods:**

The study had 3 arms: one received access to CCBT (n=301); another received CCBT plus OHC (n=302), which included a moderated internet support group; and the third received usual care (n=101). Participants in the 2 active intervention arms of the study were grouped together for analyses of CCBT use (n=603) because both arms had access to CCBT, and there were no differences in outcomes between the 2 arms. Analyses of OHC use were based on 302 participants who were randomized to that arm.

**Results:**

Several baseline patient characteristics were associated with failure to initiate the use of CCBT, including having worse physical health (measured by the Short Form Health Survey Physical Components Score, *P*=.01), more interference from pain (by the Patient-Reported Outcomes Measurement Information System Pain Interference score, *P*=.048), less formal education (*P*=.02), and being African American or another US minority group (*P*=.006). Characteristics associated with failure to initiate use of the OHC were better mental health (by the Short Form Health Survey Mental Components Score, *P*=.04), lower use of the internet (*P*=.005), and less formal education (*P*=.001). Those who initiated the use of the CCBT program but went on to complete less of the program had less formal education (*P*=.01) and lower severity of anxiety symptoms (*P*=.03).

**Conclusions:**

This study found that several patient characteristics predicted whether a patient was likely to not initiate use or discontinue the use of CCBT or OHC. These findings have clear implications for actionable areas that can be targeted during initial and ongoing engagement activities designed to increase patient buy-in, as well as increase subsequent use and the resulting success of eHealth programs.

**Trial Registration:**

ClinicalTrials.gov NCT01482806; https://clinicaltrials.gov/study/NCT01482806

## Introduction

### Background

Currently, there is a significant gap in mental health treatment. For example, almost 75% of US patients who screen positive for depressive symptoms do not participate in treatment [1]. One reason for this gap is the reliance on in-person delivery models [2]. In-person services have inherent barriers, including challenges scheduling sessions, transportation limitations, symptom exacerbations limiting the ability to travel, illness-reduced motivation, stigma, and difficulties affording out-of-pocket costs [2]. Availability of and timely access to in-person services are also limited, with >90% of psychologists and psychiatrists and 80% of professionals with a master’s degree in social work practicing exclusively in metropolitan areas [3]. In addition, treatments may have limited effectiveness. Randomized clinical trials (RCTs) of pharmacotherapy and in-person psychotherapy for mental health disorders yield effect sizes that are typically small, with 0.30 standardized mean difference [4]. This contributes to the difficulty of finding effective treatments. A study on patient-reported helpfulness of mental health treatments found that only 26.1% were helped by the first treatment they tried [5]. Persisting to a second treatment resulted in a cumulative probability of feeling helped to 51.2%. After experiencing unhelpful treatment, patients would need to persist through up to 8 providers to reach a cumulative probability of 91%, obtaining a treatment that they found helpful [5].

These characteristics of in-person treatment contribute to high rates of noninitiation, poor adherence, and discontinuation. Even in RCTs, noninitiation and premature discontinuation average approximately 30% [6]. In practice settings, almost one-third of patients referred to psychotherapies do not initiate treatment, and those who do typically discontinue during the initial sessions (up to 44% after the first session and 82% by the fifth session) [7].

eHealth approaches offer promise in addressing many of these limitations by increasing convenience, reducing the level of effort and associated motivation involved in traveling to in-person treatments, overcoming limits on the availability of practitioners and treatments, reducing the obstacles involved in initiating and switching treatments, lowering relative costs, and reducing stigma. Despite their great potential and many successes [8-10], there are commonly encountered barriers, including the levels of user digital and health literacy required, lack of confidence [11], poor designs, lack of attention to cognitive design needs [12], limited technology access, and a lack of evidence-based methods to *engage* patients with eHealth programs [13-15]. Engagement has several different meanings in the literature [16]. The view taken in this paper is that a user is engaged when he or she (1) believes that using the intervention will result in positive changes that she or he values; (2) has the motivation, confidence, and ability to initiate use; and (3) can efficiently use the technologies and eHealth program. The 3 implications of this conceptualization are that engagement is a process, usability (actual and perceived) of the technologies and their information architecture influence engagement, and intervention methods should be designed to develop *sufficient* engagement of users initially and going forward. Evidence shows that many, eHealth programs too often fail to engage users sufficiently.

Consequently, noninitiation, discontinuation, and lack of adherence can be quite high [15,17]. In recognition of the significance of engagement in the success of eHealth, and how understudied this has been, Eysenbach [17] developed the “Law of Attrition.” This law highlights inadequate buy-in by users as a fundamental methodological challenge that must be addressed if eHealth programs are to be successful in practice. In one of his examples, 99.5% of the participants discontinued MoodGym, an online, evidence-based program for depression. A recent meta-analysis of RCTs on the treatment of depressive symptoms found an estimated dropout rate of 47.8% (after adjusting for publication bias) [18]. A meta-analysis of the real-world uptake of interventions for depression, mood enhancement, and anxiety found that 12% to 79% of those who downloaded an intervention did not use the intervention once; of the remaining who did initiate use, 58% to 93% had poor adherence; and 71.4% to 99.5% discontinued before completing 40% of the intervention [19]. Eysenbach [17] argued that there is a need to develop scientific theories that provide a better understanding of the causes of such phenomena and can form the basis for creating best practices to counter them.

To gain insights into possible influences on engagement, this study used data from the Online Treatment for Mood and Anxiety Disorders in Primary Care (Online Treatment) Trial [20] to describe the characteristics of participants who did not initiate versus initiated the use of each of the 2 e-mental health interventions and discontinued versus continued using one or the other intervention. Participants were given access to a self-help computerized cognitive behavioral therapy (CCBT) program (Beating the Blues). It was provided in a collaborative care framework that included support from a “care manager.” A subgroup of participants also had access to a password-protected online health community (OHC) platform, which included moderated asynchronous internet support groups (ISGs).

Beating the Blues (CCBT), a web-based program, has been shown to be effective in treating depressive and anxiety symptoms [21-23]. The program is self-administered and consists of a brief 10-minute introductory video followed by eight 50-minute-long interactive modules. The modules’ topics are (1) problem definition and pleasurable events; (2) automatic thoughts; (3) thinking errors and distraction; (4 and 5) challenging unhelpful thinking; (6) core beliefs; (7) attributional style; and (8) review, action planning, and conclusion. Each module uses text, audio, audiovisual clips, and “homework” assignments designed to impart basic cognitive behavioral therapy techniques that target depression and anxiety. These 8 modules must be completed sequentially.

ISGs can improve users’ illness knowledge, coping skills, emotional support, connectedness, self-efficacy, and mental health [24-27]. Studies on users of web-based peer support have found that more frequent use results in more mental health benefits [28]. To explore the potential of peer support to enhance the effectiveness of CCBT, which is fully self-guided and does not offer the option for interaction with peers or providers, we included OHC. This provided access to several ISGs that the participants could use. However, nontherapeutic interactions can occur in these groups, which may result in negative consequences for users [29]. Therefore, it is important to use methods to keep the interactions supportive. The ISGs in this study had a moderator to manage content, facilitate supportive interactions, and amplify potential benefits [24].

### Objective

The objective of this study was to identify patient baseline characteristics that were associated with noninitiation, poor adherence, discontinuing, or continuing to use each of 2 eHealth treatments for anxiety and depression, CCBT and OHC. This study conducted secondary analyses of data collected from the parent randomized controlled trial, Online Treatment for Mood and Anxiety Disorders in Primary Care [20]. The parent study provided CCBT and OHC to primary care patients with elevated depressive or anxiety symptoms.

## Methods

### Ethics Approval

The institutional review board of the University of Pittsburgh approved all study procedures (reference number: 20030187), and all participants provided written informed consent.

### Overview

This study is a secondary analysis of data from the parent randomized controlled trial, the Online Treatment for Mood and Anxiety Disorders in Primary Care (Online Treatment) trial that examined the impact of access to 2 e-mental health interventions or their primary care physician’s (PCP’s) usual care (UC) on clinical outcomes [20]. The institutional review board of the University of Pittsburgh approved the study protocol, and all participants provided written informed consent.

Briefly, the parent trial recruited patients from 26 primary care offices affiliated with the University of Pittsburgh Medical Center, which shared a common electronic medical record system. A PCP received an electronic medical record system reminder about the study at the time of the clinical encounter for all patients aged 18 to 75 years with a diagnosis of depression, anxiety, generalized anxiety, or panic disorder. If the patient agreed to a study referral, they were contacted by a study recruiter via telephone to confirm protocol eligibility.

### Participants

Eligible patients needed to have at least a moderate level of depressive or anxiety symptoms with a score 10 or greater on the 9-item Patient Health Questionnaire (PHQ-9) [30] or the 7-item Generalized Anxiety Disorder Scale [31]; reliable access to the internet, email, and telephone; and no alcohol dependence (as determined by the Alcohol Use Disorders Identification Test) [32], active suicidality, or other serious mental illness. Research assessors then administered via telephone the baseline battery and collected information on patients’ self-reported race, sex, and other sociodemographic characteristics. This study was registered to ClinicalTrials.gov under trial registration number NCT01482806.

### Randomization Procedure

Following the baseline assessment, patients were randomized in a 3:3:1 ratio to (1) access to a self-guided CCBT program (CCBT-only [Beating the Blues]; n=301); (2) CCBT plus access to a password-protected OHC platform that included moderated ISGs (CCBT+OHC; n=302); or (3) UC of their PCP (n=101). The present report only used data from participants randomized to the CCBT-only and CCBT+OHC intervention groups (N=603).

### Interventions

#### Computerized Cognitive Behavioral Therapy

The Beating the Blues web-based program (described earlier) served as the CCBT intervention [33]. CCBT was provided in a collaborative care framework via support from a care manager. The care managers encouraged participants to complete a module every 1 to 2 weeks, but the participants were free to proceed at their own pace.

#### Online Health Community

The OHC was password-protected and featured collections of links to external resources (eg, crisis hotlines, “find-a-therapist” sites, local US $4 generic pharmacy programs) and brief YouTube videos on stress management, sleep hygiene, meditation, exercise benefits, and nutrition. In addition, the participants could interact with one another on a variety of moderated ISGs. Each ISG was dedicated to a specific topic [34]. Unlike the CCBT, the OHC was designed so that participants had freedom of choice regarding the resources they used.

A study investigator logged into the ISGs daily to review new posts and monitor for the presence of suicidal thoughts or potentially inappropriate content. In addition, the OHC had a moderator who oversaw all communications by participants. The moderator was a care manager (described below) for this study. To promote participants’ ongoing involvement with the OHC a variety of strategies were used: (1) weekly emails from the moderator that highlighted new discussions, new content, or self-management tips; (2) status indicators on participants’ profiles and comments they posted to a discussion (eg, stars and “likes”); (3) automatically generated email notifications of new ISG activities, for example, when someone replied to a participant’s ISG post or comment; (4) automated highlighting of recent comments on participants’ home pages, which were personalized to their ISG profile and past comments; (5) invited participants as guest moderators; and (6) initiating contests (eg, scavenger hunts, respond to emails or posts). During regular care management contacts, the care manager directed the participants to pertinent content on the OHC. For safety, at least one study clinician logged into the ISGs daily to monitor user-generated posts and comments, and the participants were able to “flag” potentially inappropriate content for removal.

#### Collaborative Care Framework: Care Managers Promote Engagement, Ongoing Involvement, and Effectiveness

Both eHealth programs were delivered via a collaborative care strategy. The 2 care managers had a bachelor’s or master’s degree in the psychology field and had worked on conducting mental health research with human participants. After randomization, a care manager dedicated to each intervention arm contacted the patient for an introductory telephone call and provided guidance in the setup of access to the CCBT program and the OHC, if applicable. During the 6-month intervention, participants were encouraged to complete a CCBT module every 1 to 2 weeks and were provided reminders, if necessary, encouragement on their progress, and personalized feedback. Participants had the option to contact a care manager with questions or for assistance. Care managers, irrespective of eHealth program usage, monitored patients’ progress, symptoms, use of the eHealth programs, and telephoned those who either were not doing well, as indicated by their scores on the PHQ-9, or had failed to log onto the CCBT or OHC regularly to inquire why. They entered all contacts and information gathered into an electronic registry that was used to track each patient’s progress and guide care managers through their contacts. If a patient’s symptoms did not improve or worsen, they contacted her or him via email or telephone (depending on the patient’s preference) to discuss additional treatment options, depending on the patient’s preference. At weekly case review meetings, with the assistance of the electronic registry, the care managers presented their patients to the study clinical team, which consisted of a PCP, psychiatrist, and psychologist-study coordinator (study team). In addition to providing patients with suggestions for general lifestyle adjustments, including social engagement, exercise, adequate sleep, and nutrition suggestions, the study team also recommended initiation or modification of antidepressant or anxiolytic pharmacotherapy based on patients’ symptom response and treatment preferences and referral to a mental health specialist when a patient had either complex psychosocial issues or was not responding to treatment. Following the case review, the care managers discussed recommendations with the patient and then notified the patient’s PCPs of progress and treatment suggestions. PCPs remained responsible for the treatment and were free to continue or modify the patients’ treatments.

### Assessments

Blinded telephone assessors administered the assessment battery at baseline, 3 months, 6 months (end of interventions), and 12 months to assess the durability of the interventions. It included the administration of the 12-Item Short Form Health Survey (SF-12) assessment of SF-12 Mental Component Summary (MCS; SF-12 MCS) and Physical Component Summary (PCS; SF-12 PCS) scores, composed of 6 items each from the SF-12 to measure these 2 aspects of health-related quality of life [35]; the PHQ-9, a fixed-length Patient-Reported Outcomes Measurement Information System (PROMIS) measure to assess the severity of depressive symptoms [30]; the Generalized Anxiety Disorder 7- item scale to assess anxiety severity [31]; the 8-item fixed-length PROMIS-PI to assess pain interference, that is, the degree to which pain interferes with an individual’s physical, mental, and social daily activities [36]; and the Primary Care Evaluation of Mental Disorders to provide diagnoses of depressive and anxiety disorders [37]. In addition, an 11-item shortened version of the Pew Internet Use questionnaire was administered at baseline to assess participants’ use of the internet; server logs were abstracted to measure the use of the CCBT and OHC programs; and care managers’ electronic registry was used to assess the number of intervention emails and telephone contacts.

### Statistical Analyses

As the purpose of this study was to identify subgroups of participants based on their CCBT or OHC use patterns, we limited the analyses to the CCBT alone and CCBT+OHC treatment arms (N=603). As documented in a previous study, both were more effective than PCPs’ UC for depression and anxiety but were similarly effective to each other (ie, offering access to the OHC produced no added reduction in depressive or anxiety symptoms over CCBT alone) [20]. Consequently, for CCBT analyses, we combined CCBT users from both arms (N=603).

We first classified the participants according to whether they initiated the use of the CCBT program. Participants who logged in at least once were categorized as having initiated CCBT use, whereas participants who never logged in comprised the noninitiation group. We compared baseline sociodemographic, clinical, and functional status measures by initiation status using *t* tests for continuous data and chi-square tests for categorical data. Fisher exact test was used for categorical measures when the expected cell counts were less than 5. Participants were then stratified by whether they “continued” the use of CCBT (yes or no), as those who completed at least one CCBT module. Those who did not complete the first CCBT module were categorized as “discontinued” use. Baseline characteristics were compared between these 2 groups (ie, continued and discontinued use) using *t* tests and chi-squared tests (or Fisher exact test, as appropriate). On the basis of previous work with this data set that found differences in the effects of CCBT between African American participants and White participants [38], we also examined whether there might be differences in the use patterns.

Participants in the CCBT+OHC study arm were then classified as initiation or noninitiation of OHC. Those who logged in at least once were considered to have initiated OHC use, whereas those who never logged in were categorized as noninitiators of OHC use. The baseline measurements were compared between the 2 groups. Continuation of OHC use was evaluated by stratifying OHC participants into 3 mutually exclusive categories: those who logged in only once, those who logged in 2 to 3 times, and those who logged in more than 3 times during the 6-month intervention phase. The baseline characteristics were compared across these groups. Finally, continuation of OHC use was classified by the number of months during which participants logged in to the OHC: logged in during 1 month only, logged in during 2 or 3 months, and logged in over a period of more than 3 months during the intervention phase. The participant characteristics were compared between the groups. All statistical analyses were performed using R version 3.6.3 (R Foundation for Statistical Computing). A significance level of α of .05 was assumed, and no adjustments were made for multiplicity.

## Results

### Participant Description

Participants (N=603) in this study had a mean age of 42.8 (SD 14.2) years, were 79.6% (n=480) female, 82.8% (n=499) were White, 82.4% (n=497) had some college education, and 69.8% (n=421) were employed (Table 1). At baseline, they reported a mean SF-12 PCS of 50.9 (SD 12.3), which is similar to that of the general US population, 50 (SD 10) [35]. The mean SF-12 MCS was 31.5 (SD 8.9), which is considerably lower than the 50 (SD 10) for the general US population [35]. The participants’ mean PROMIS depression and anxiety scores were 62.3 (SD 12.3), and 65.9 (SD 12.3), respectively. Both indicate a higher severity of symptoms than the general US population, which had a mean score of 50 [39]. The 2 intervention arms did not differ in their baseline sociodemographic or clinical characteristics by random treatment assignment (all *P*≥0.4, previously published) [20].

**Table 1:** Baseline characteristics for intervention initiators and noninitiators.

Measure and category				Total (n=603)		Computerized cognitive behavioral therapy								Online health community						
						Initiation (n=521)		Noninitiation (n=82)		*P* value			Initiation (n=228)			Noninitiation (n=74)			*P* value	
**Demographics**																				
	Age (y), mean (SD)			42.8 (14.2)		42.8 (14.2)		43.1 (14.4)		.84			42.2 (14)			43.9 (15.5)			.38	
	**Sex, n (%)**										.94									.31
		Female	480 (79.6)		415 (79.7)		65 (79.3)					182 (79.8)			63 (85.1)					
		Male	123 (20.4)		106 (20.3)		17 (20.7)					46 (20.2)			11 (14.9)					
	**Race, n (%)**										.006									.09
		White	499 (82.8)		441 (84.6)		58 (70.7)					188 (82.5)			54 (73)					
		African American	91 (15.1)		69 (13.2)		22 (26.8)					34 (14.9)			19 (25.7)					
		Other	13 (2.2)		11 (2.1)		2 (2.4)					6 (2.6)			1 (1.4)					
	**Living situation, n (%)**										.06									.60
		Alone with child	82 (13.6)		64 (12.3)		18 (22)					23 (10.1)			11 (14.9)					
		Alone no child	114 (18.9)		100 (19.2)		14 (17.1)					45 (19.7)			15 (20.3)					
		Living together with child	106 (17.6)		89 (17.1)		17 (20.7)					45 (19.7)			11 (14.9)					
		Living together with no child	300 (49.8)		267 (51.3)		33 (40.2)					115 (50.4)			37 (50)					
	**Working, n (%)**										.85									.15
		Employed	421 (69.8)		363 (69.7)		58 (70.7)					159 (69.7)			45 (60.8)					
		Other	182 (30.2)		158 (30.3)		24 (29.3)					69 (30.3)			29 (39.2)					
	**Education, n (%)**										.02									<.001
		High school or less	106 (17.6)		84 (16.1)		22 (26.8)					25 (11)			23 (31.1)					
		Attended college but did not receive a 4-year degree (also business or technical school)	216 (35.8)		184 (35.3)		32 (39)					86 (37.7)			24 (32.4)					
		College degree or higher	281 (46.6)		253 (48.6)		28 (34.1)					117 (51.3)			27 (36.5)					
**Clinical characteristics**																				
	Short Form Health Survey Physical Components Score, mean (SD)			50.9 (12.3)		51.4 (12.1)		47.7 (12.8)		.01			51.3 (12.5)			50.3 (11.8)			.56	
	PROMIS^a^ pain interference, mean (SD)			31.5 (8.9)		31.3 (8.8)		32.4 (10)		.30			31.1 (8.8)			33.6 (10.9)			.04	
	Short Form Health Survey Mental Components Score, mean (SD)			17.8 (9.3)		17.5 (9.3)		19.7 (9.4)		.048			17.2 (9.2)			18.8 (9.7)			.19	
	PROMIS depression, mean (SD)			62.3 (6.2)		62.2 (6.4)		62.6 (4.8)		.55			62.2 (6.3)			61.5 (6.1)			.41	
	PROMIS anxiety, mean (SD)			65.9 (6.1)		65.9 (6)		65.7 (6.4)		.82			66 (6.1)			65.2 (6.2)			.35	
	PROMIS sleep impairment, mean (SD)			24.1 (5.8)		24 (5.8)		25.1 (5.6)		.12			24.2 (6)			24.6 (5.5)			.68	
**Mobile and internet use, n (%)**																				
	**Mobile phone use**										.82									.32
		Yes	405 (67.2)		349 (67)		56 (68.3)					162 (71.1)			48 (64.9)					
		No	198 (32.8)		172 (33)		26 (31.7)					66 (28.9)			26 (35.1)					
	**Nonwork internet use**										.13									.005
		Never or rare	46 (7.6)		36 (6.9)		10 (12.2)					12 (5.3)			11 (14.9)					
		Occasional	90 (14.9)		75 (14.4)		15 (18.3)					30 (13.2)			15 (20.3)					
		Consistent	467 (77.4)		410 (78.7)		57 (69.5)					186 (81.6)			48 (64.9)					
	**Work internet use**										.38									.19
		Never or rare	282 (46.8)		239 (45.9)		43 (52.4)					102 (44.7)			42 (56.8)					
		Occasional	29 (4.8)		24 (4.6)		5 (6.1)					10 (4.4)			3 (4.1)					
		Consistent	292 (48.4)		258 (49.5)		34 (41.5)					116 (50.9)			29 (39.2)					

^a^PROMIS: Patient-Reported Outcomes Measurement Information System.

### Noninitiation Versus Initiation of CCBT Use

When pooling CCBT users from the CCBT-only and CCBT+OHC arms, 603 participants had access to the CCBT program. During the 6-month intervention, 13.6% (82/603) did not initiate the use of the program (Table 1). Those who did not initiate use were more likely to have a lower (worse) SF-12 PCS (*P*=.01), higher (worse) PROMIS Pain Interference measure (*P*=.048), and less likely to have a 4-year college degree (*P*=.02). The rate of noninitiation was 24% (22/91) for African American participants, 11.6% (58/499) for White participants, and 15% (2/13) for participants of “other” races (*P*=.006).

### Discontinue Versus Continue Use of CCBT

Of those who initiated use (n=521), 97% (504/521) completed the first module (Table 2), and 12.9% (65/504) discontinued on completing module 1. If a patient was going to complete the first module, she or he did so during the first 3 months (495/504, 98%). Only an additional 9 (1.8%) participants completed the first module in the subsequent 3 months (data not shown).

**Table 2:** Baseline characteristics for sample, intervention users, and nonusers.

Categories, measure, and category				Total (n=603)		CCBT^a^ user^b^ (n=504)		CCBT nonuser^c^ (n=99)		*P* value			OHC^d^ user (n=228)			OHC nonuser (n=74)			*P* value	
**Demographics**																				
	Age (y), mean (SD)			42.8 (14.2)		43 (14.2)		42 (14.4)		.54			42.2 (14)			43.9 (15.5)			.38	
	**Sex, n (%)**										.62									.31
		Female	480 (79.6)		403 (80)		77 (77.8)					182 (79.8)			63 (85.1)					
		Male	123 (20.4)		101 (20)		22 (22.2)					46 (20.2)			11 (14.9)					
	**Race, n (%)**										.001									.09
		White	499 (82.8)		429 (85.1)		70 (70.7)					188 (82.5)			54 (73)					
		African American	91 (15.1)		64 (12.7)		27 (27.3)					34 (14.9)			19 (25.7)					
		Other	13 (2.2)		11 (2.2)		2 (2)					6 (2.6)			1 (1.4)					
	**Living situation, n (%)**										.048									.60
		Alone with child	82 (13.6)		60 (11.9)		22 (22.2)					23 (10.1)			11 (14.9)					
		Alone with no child	114 (18.9)		98 (19.5)		16 (16.2)					45 (19.7)			15 (20.3)					
		Living together with child	106 (17.6)		88 (17.5)		18 (18.2)					45 (19.7)			11 (14.9)					
		Living together with no child	300 (49.8)		257 (51.1)		43 (43.4)					115 (50.4)			37 (50)					
	**Working, n (%)**										.65									.15
		Employed	421 (69.8)		350 (69.4)		71 (71.7)					159 (69.7)			45 (60.8)					
		Other	182 (30.2)		154 (30.6)		28 (28.3)					69 (30.3)			29 (39.2)					
	**Education, n (%)**										<.001									<.001
		High school or less	106 (17.6)		76 (15.1)		30 (30.3)					25 (11)			23 (31.1)					
		Attended college but did not receive a 4-year degree (also business or technical school)	216 (35.8)		177 (35.1)		39 (39.4)					86 (37.7)			24 (32.4)					
		College degree or higher	281 (46.6)		251 (49.8)		30 (30.3)					117 (51.3)			27 (36.5)					
**Clinical characteristics, mean (SD)**																				
	Short Form Health Survey Physical Components Score			50.9 (12.3)		51.4 (12.1)		48.4 (12.7)		.03			51.3 (12.5)			50.3 (11.8)			.56	
	PROMIS^e^ pain interference			17.8 (9.3)		17.5 (9.3)		19.5 (9.4)		.049			17.2 (9.2)			18.8 (9.7)			.19	
	Short Form Health Survey Mental Components Score			31.5 (8.9)		31.3 (8.7)		32.2 (9.9)		.36			31.1 (8.8)			33.6 (10.9)			.04	
	PROMIS depression			62.3 (6.2)		62.2 (6.5)		62.6 (5)		.56			62.2 (6.3)			61.5 (6.1)			.41	
	PROMIS anxiety			65.9 (6.1)		65.8 (6.1)		65.9 (6.2)		.92			66 (6.1)			65.2 (6.2)			.35	
	PROMIS sleep impairment			24.1 (5.8)		24 (5.8)		24.8 (5.6)		.22			24.2 (6)			24.6 (5.5)			.68	
**Mobile and internet use**																				
	**Mobile phone use, n (%)**										.56									.32
		Yes	405 (67.2)		336 (66.7)		69 (69.7)					162 (71.1)			48 (64.9)					
		No	198 (32.8)		168 (33.3)		30 (30.3)					66 (28.9)			26 (35.1)					
	**Nonwork internet use, n (%)**										.12									.005
		Never or rare	46 (7.6)		35 (6.9)		11 (11.1)					12 (5.3)			11 (14.9)					
		Occasional	90 (14.9)		71 (14.1)		19 (19.2)					30 (13.2)			15 (20.3)					
		Consistent	467 (77.4)		398 (79)		69 (69.7)					186 (81.6)			48 (64.9)					
	**Work internet use, n (%)**										.22									.19
		Never or rare	282 (46.8)		231 (45.8)		51 (51.5)					102 (44.7)			42 (56.8)					
		Occasional	29 (4.8)		22 (4.4)		7 (7.1)					10 (4.4)			3 (4.1)					
		Consistent	292 (48.4)		251 (49.8)		41 (41.4)					116 (50.9)			29 (39.2)					

^a^CCBT: computerized cognitive behavioral therapy.

^b^Completed the first CCBT module.

^c^Did not complete the first CCBT module.

^d^OHC: online health community.

^e^PROMIS: Patient-Reported Outcomes Measurement Information System.

Before completing the first module, 16.4% (99/603) discontinued CCBT. These participants were more likely at baseline to be a single parent living with their child, have a lower SF-12 PCS, have a higher PROMIS pain interference with life score, and less likely to have a 4-year college degree. African American participants were more likely to discontinue use than White participants (Figure S1 in Multimedia Appendix 1). Just over half (261/504, 51.8%) discontinued CCBT before completing 7 or 8 modules. Those who completed more modules were less likely to be mobile phone users, more likely to have a 4-year college degree, and more likely to have a higher severity of anxiety symptoms at baseline. There were no differences in baseline characteristics between those who completed module 7, started but did not finish module 8, or completed module 8. For analysis purposes, this group was defined as the completers of the CCBT. Those who initiated use but discontinued before completing were 64% (41/64) African Americans and 50.6% (217/429) White (*P*=.04).

### Noninitiation Versus Initiation of OHC Use

During the 6-month intervention, 24.5% (74/302) of the participants with OHC access did not initiate use (Table 1). This group, when compared with those who initiated use, was less likely to have a 4-year college degree (*P*<.001), use the internet outside of work (*P*=.005), and more likely to have a better SF-12 MCS (*P*=.04). The rate of noninitiation was higher for African American participants (19/53, 36%) than for White participants (54/242, 22.3%; *P*=.04). Patients who waited to initiate use until sometime after the first month of having access had a higher PROMIS pain interference with life score (*P*=.03), poorer SF-12 PCS (*P*=.04), and were less likely to use the internet outside of work (*P*=.003).

### Discontinue Versus Continue Use of the OHC

Of the 75% (n=228) of patients who initiated the use of the OHC during the 6-month intervention, 19% (43/228) discontinued use after logging in only once, 25% (56/228) discontinued use after logging in 2 to 3 times, and 56.6% (129/228) logged in 4 or more times (Figure S2 in Multimedia Appendix 1). Those who logged in fewer times tended to be younger (*P*=.03) and African American (*P*=.005).

## Discussion

### Principal Findings

In this study, the vast majority of patients initiated the use of the eHealth programs. Over half of the participants discontinued using CCBT before completion, and 43% (99/228) logged into the OHC 3 or fewer times. These latter groups provide an opportunity to explore areas where improvements in engagement methods and eHealth interventions could be made.

### Noninitiation of CCBT and OHC

Patients who did not initiate the use of the CCBT or OHC programs were more likely to have less formal education, and those who did not initiate the use of the OHC also tended to be less frequent internet users. These findings are consistent with the conclusion that those who have less experience with technology may be less savvy or confident with technology, and that there is more reluctance to initiate use of web-based programs. Reluctance due to one’s level of digital or health literacy is addressable at the start by helping to develop greater confidence in the technologies involved in the interventions and the ability of the contents to be effective. In this study, all participants needed to have access to the necessary technologies for participation; however, the care managers also offered telephone assistance with technical difficulties with the e-mental health programs. Some may have felt stigmatized asking for help, particularly with their personal technologies; felt that phone guidance would not work; or felt that this was not an offer to assist with using the programs or their personal technologies but for technical issues outside of their control.

Poor physical well-being, as indicated by lower SF-12 PCS scores or higher pain interference with life, also reduced the likelihood of patients initiating use. This raises several possibilities. First, it may be that these patients had more difficulty using the technologies and websites due to physical limitations and associated pain or they may have had a reduced ability to concentrate on the contents. Another possibility is that they were less enthusiastic about the interventions because they were not directly focused on physical health issues; thus, they were perceived as not addressing their high-priority need. However, it has been well established that chronic pain, depression, anxiety, and somatic amplification co-occur [40]. Such issues would be important to address during early engagement activities designed to both develop confidence in the ability of the interventions to help and to assess and address reasons for patients’ hesitancy to use the interventions.

Those who did not initiate the use of the interventions during the first month of the trial were unlikely to later. If the methods designed to develop engagement with the interventions did not sufficiently influence a patient during the initial weeks, they were unlikely to have a subsequent effect. This may help identify an area where certain patients could benefit from earlier or additional methods to increase their engagement.

### Discontinuation of CCBT

Patients who did not complete the first CCBT module were likely to be less technology savvy, have poorer physical functioning, have higher pain interference with life, and have less severe anxiety symptoms at baseline. Mental health burden, comfort with technology, and physical functioning were recurring influences on patient initiation and discontinuation. Others have also found that an increased mental health burden is associated with greater use of e-mental health interventions for anxiety and depressive symptoms [41]. A counterintuitive finding was that those less likely to be mobile phone users were more likely to complete a higher number of CCBT modules. This could be because they had less access to web-based resources, which made the offered treatments a more unique and valued opportunity to receive mental health services.

### Discontinuation of CCBT and OHC

Given that over half of the participants discontinued the CCBT program before completion, this may indicate the need for additional ongoing methods to maintain users’ buy-in. It should be noted that CCBT did have a positive effect on clinical outcomes compared with UC [20], indicating that to receive benefit, at least partial benefit, it was not necessary to complete the program. Some patients may have felt that the program was no longer addressing their needs or that they improved sufficiently and discontinued [42]. This raises the issue of whether setting patients’ treatment expectations, for example, for when full benefit has been gained, would influence discontinuation and improve outcomes for those who leave before completion. Examining patients’ reasons for discontinuing would provide patient-centered insights on this issue and potentially how the interventions could be tailored as needs change during use.

African American participants who initiated the use were more likely than White participants to discontinue OHC use after only one login and more likely to discontinue CCBT use before completing the program. Taken together, these findings indicate that more could be done to facilitate the engagement of African Americans, and possibly other minority groups. This may also indicate that content adaptations could improve the intervention’s fit for diverse groups [38]. Digital literacy and internet connectivity, which include skills, confidence, connectivity and its ongoing affordability, level of access, devices, training, and technical support, have been called the “super social determinants of health” because they influence all other social determinants of health [43]. The study examined only a few social determinants of health, for example, employment and education. It is likely that other social determinants of health influenced these findings, and this is a key area for future investigations.

### The Importance of Strategies to Facilitate Engagement

Only a minority of patients did not initiate the use of e-mental eHealth interventions, and almost half of them completed CCBT. This argues for the effectiveness of the engagement offered by the ongoing collaborative care strategies used. This is supported by the findings from 2 earlier studies on CCBT. The original study that established the efficacy of CCBT provided in-person treatment at a research office [21]. One module was completed during each visit. Each user was provided with 1:1 supervision from a practice nurse. The nurse ensured that each participant interacted successfully with the computer and treatment program. A subsequent study examined the web-based home delivery of CCBT. Investigators found no effect of CCBT when compared with UC [44]. In this study, although participants were called weekly at their homes by a research staff member who encouraged continued program use, the amount of contact over the 4-month study period was an average of only 23 seconds per week.

These findings support the potential of providing eHealth programs via collaborative care and supported and “guided” models [45]. They also point to the potential of including additional and improved methods to facilitate engagement. Highly effective engagement practices, research findings, and theoretical models have identified several best practices to engage patients with interventions. These identify at least 5 characteristics of an intervention model that facilitate strong engagement [14,17,46-49]. One is compatibility. Engagement activities should establish how the intervention will be personally meaningful and able to meet the needs a user finds important. For example, it will facilitate better illness management or an improved ability to socialize or participate in work. In this study, it may have been helpful if a patient’s PCP or care manager had introduced the programs and engaged in shared decision-making to identify specific benefits to the patient. In addition, participants may have considered the interventions more of a study than personalized treatment because they were not presented by their PCP. Another characteristic is relative advantage. This is the level of belief that the effort and resources involved will provide sufficient added value over alternatives. In total, 2 alternatives that patients commonly consider are not initiating the treatment and/or discontinuing. The study’s CCBT program may have been too rigid for some patients, as they could not skip a module or tailor the program to their immediate needs. This feedback was provided to the care managers. Several existing and recent eHealth programs have developed more flexible approaches that allow users to move through the program and tailor the presentation according to their current needs and previous experience with mental health treatment [50-52]. However, many eHealth interventions with fixed presentations are highly effective [53]. Creating approaches that can accommodate diverse and even changing needs of patients may help support broader engagement. A third characteristic is the complexity of the intervention. This is the extent to which the intervention and required technologies are intuitive to use either before or after training. This can be especially important for users without sufficient digital or health literacy, and in-person training may be beneficial. Fourth characteristic is observability. This is the extent to which it will be easy for users to see improvements in issues that are important to them because of their use of the intervention. This may have manifested in 2 opposite ways. Some may have perceived initial improvement and thought that it was all that could be expected. Others may have felt hampered by the rigidity of the CCBT program because patients may have had to complete modules that did not seem relevant to them; thus, they did not see added benefit in continuing the program. Both these factors could have led to discontinuation. The fifth characteristic is supportive accountability. This includes support from a human coach or care manager who can provide guidance and develop accountability on the part of users. This can increase adherence. In this study, the personalized and continuous support provided via the collaborative care strategy, and knowing that care managers would call to check on progress and issues around completing homework, likely contributed to the high initiation and ongoing use rates. The ability of such “guidance” to improve initiation and adherence by some users has been documented [45,51]. This argues for the considerable advantage of guided collaborative care models such as was used in this study.

### Conclusions

These analyses produced several findings: those with greater mental health needs had a greater predisposition to initiate and continue use, those with poorer physical well-being were less likely to initiate use, and comfort with technologies seemed to influence patients’ likelihood of initiating and continuing use. These findings support the conclusion that methods are needed to build engagement with eHealth interventions that can be tailored to these as well as other specific needs of patients.

Although discontinuation has been explored to a somewhat greater extent in the eHealth literature, less attention has been devoted to noninitiation, and neither has been thoroughly investigated. The findings highlight a potentially important need for additional studies of both noninitiation and discontinuation and the relevance of these 2 phenomena to the types of personalized engagement and ongoing support methods that could benefit users and help address the gaps that lead to Eisenbach’s [17] Law of Attrition.

## Appendix

Multimedia Appendix 1 Number of computerized cognitive behavioral therapy program modules completed and number of logins to the online health community platform.

## Abbreviations

CCBT: computerized cognitive behavioral therapy
ISG: internet support group
MCS: Mental Components Score
OHC: online health community
PCP: primary care physician
PCS: Physical Components Score
PHQ-9: 9-item Patient Health Questionnaire
PROMIS: Patient-Reported Outcomes Measurement Information System
RCT: randomized clinical trial
SF-12: Short Form Health Survey
UC: usual care
