# Supplementary figures and images for: Predictors of Use and Drop Out From a Web-Based Cognitive Behavioral Therapy Program and Health Community for Depression and Anxiety in Primary Care Patients: Secondary Analysis of a Randomized Controlled Trial
Source: JMIR Ment Health. 2024 Jan 17;11:e52197. doi: 10.2196/52197 (PMC10836415; doi:10.2196/52197)

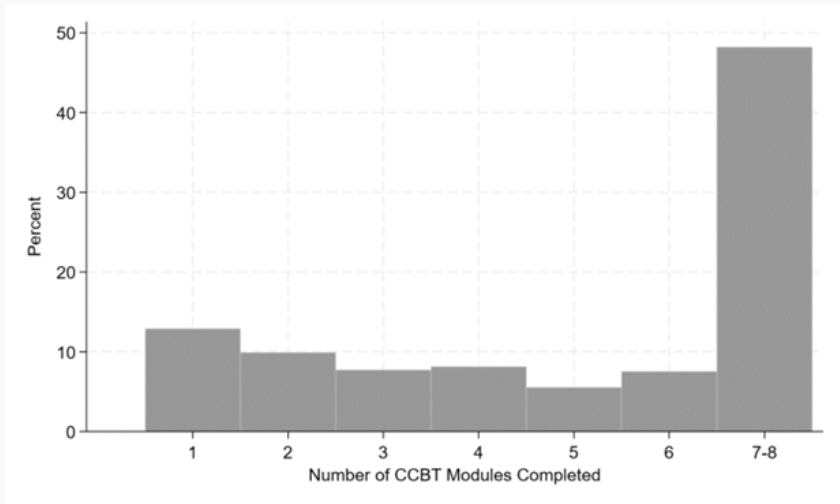


Figure S1


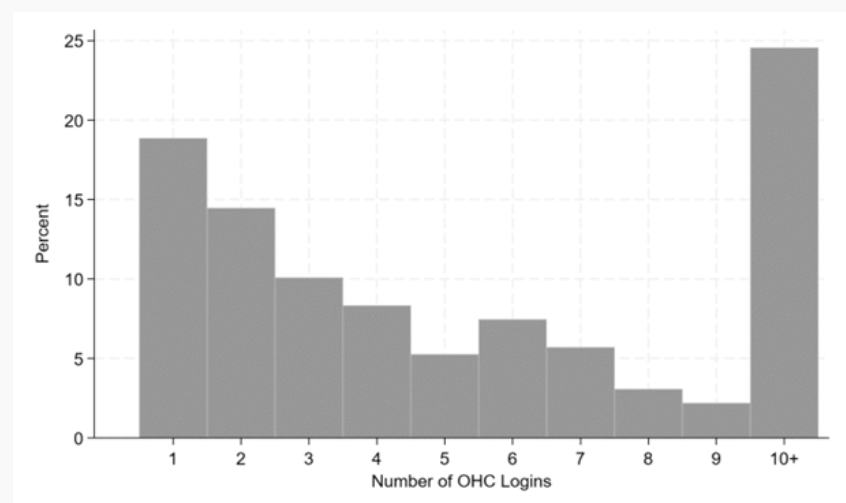


Figure S2

Supplement: Multimedia Appendix 1 [file mental_v11i1e52197_app1.docx]
